# Topological Descriptors of Colorectal Cancer Drugs and Characterizing Physical Properties Via QSPR Analysis

**DOI:** 10.1155/ianc/5512172

**Published:** 2025-02-15

**Authors:** Sumiya Nasir

**Affiliations:** Department of Mathematics and Natural Sciences, College of Sciences and Human Studies, Prince Mohammad Bin Fahd University, Khobar 31952, Saudi Arabia

**Keywords:** colorectal cancer drugs, QSPR models, topological descriptors

## Abstract

Topological descriptors and QSPR analysis are statistical techniques that are highly beneficial for analyzing various physical and chemical characteristics of molecular graphs without necessitating expensive and time-consuming laboratory experiments. The topological descriptor alters the compound to a number and helps in finding physicochemical properties. It more correctly reproduces the theoretical properties of drugs. In this article, the author investigated colorectal drugs capecitabine, leucovorin, tipiracil hydrochloride, etc. and implemented QSPR analysis. Physical qualities such as molar volume, complexity, polarity, and refractivity are the subject of the current study. The outcomes of this study allow for more effective physical property prediction through the use of QSPR models. First, we calculate Tds and secondly perform QSPR analysis. Current work on TIs and QSPR modeling shows a good correlation with physical properties. Moreover, estimated drug results depict and predict the physical properties in an efficient way.

## 1. Introduction

Colorectal cancer (CRC) is the third most frequent cancer and the leading cause of cancer death in the United Kingdom. Despite major improvements in our understanding of early detection and prevention, the disease ranks third in terms of cancer-related deaths [1]. In Asia, CRC is the fourth most common cancer-related cause of death, and its incidence is rising. CRC is one of the main causes of cancer fatalities in Europe [2] and Asia. Epidemiological data have revealed that several aspects with family history, excessive meat ingesting, alcohol use, and genetic changes impact the risk of colorectal neoplasia [3]. In CRC, cells in the colon proliferate out of control. The remaining waste material from the colon enters the rectum, the stomach's last six inches of digestive tract. The depth to which a CRC penetrates the wall and if it has progressed outside the colon or rectum determine the stage of the disease. However, the exact mechanism underlying colorectal carcinogenesis is still not known. CRC may have both hereditary and environmental causes, unlike other complicated diseases [4]. With 783,000 new cases per year, CRC is a serious health issue that accounts for 9% of all cancers in the globe [5]. It peaked in the United States in 1994 with 131,000 new cases and 57,000 fatalities. There is growing evidence that CRC can spread to numerous organs and that metastatic illness is the leading cause of death in CRC patients. The liver is the CRC metastases' preferred target [6–8]. The second most frequent disease in the world and the second largest cause of cancer death, sporadic CRC accounts for 75% of all CRC cases [9]. Adenoma is the lesion that occurs first in a series that could last 10–15 years before CRC. If men and women are taken into account jointly, CRC is the most prevalent malignancy in the world, with more than 1,200,000 new cases per year. North America, Europe, Australia, and New Zealand have the greatest incidence rates. Moreover, lung cancer is the second most common cause of cancer-related fatalities in these populations. Since premalignant polyps account for more than 85% of tumor emergence, CRC may be prevented. Therefore, the goal of CRC screening is to lower mortality by detecting people who have neoplastic lesions that are presymptomatic and who may need additional testing and treatment. Testing procedures should be affordable, regarded as appropriate, sensitive, focused, and secure. However, none of the suggested assays for CRC screening that are currently available meet these conditions [10]. A CRC-related death occurs every 9 minutes, according to the reports. In addition, it ranks third among the most prevalent malignancies in the WHO EMRO region, behind lung and breast cancer in women and cervical cancer in males [11,12].

**Figure 1:**
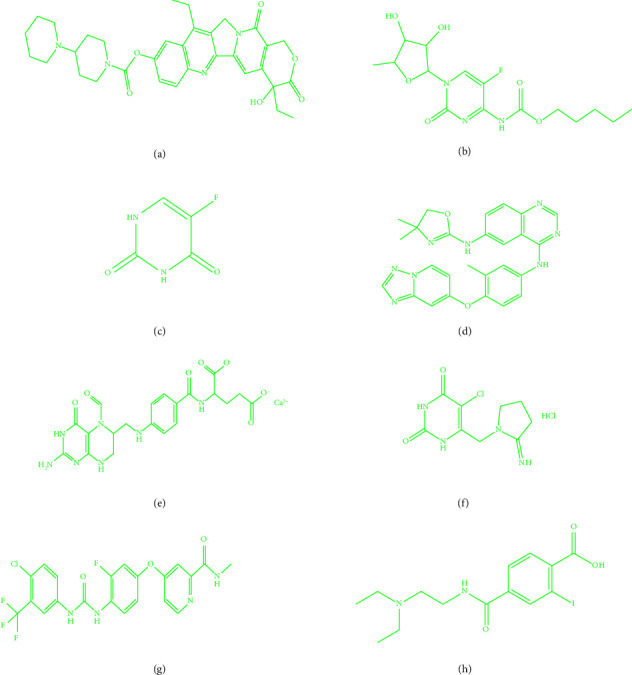
Colorectal cancer drugs. (a) Irinotecan, (b) capecitabine, (c) fluorouracil, (d) tucatinib, (e) leucovorin,(f) tipiracil hydrochloride, (g) regorafenib, and (h) bevacizumab.

CRC slays a number of individuals. Researchers create and study novel medications. Their creation is a difficult job because of costly, time-consuming, and incredibly tough. Many drugs trial is imposed to stop this deadly disease, and numerous drug tests are imposed to combat lethal disease. It demands swift finding and medication that will device the disease. Eight drugs' medicines irinotecan, capecitabine, fluorouracil, leucovorin, tipiracil hydrochloride, regorafenib, tucatinib, and bevacizumab are harmless and are more effective in nature for well-being community.  Figure 1 displays the aforementioned drugs. All Tds are important and show a noteworthy role in chemical graphs as they can expose and predict hidden molecular graph properties. Tds not only has applications in science such as cheminformatics and bioinformatics but also has a substantial role in quantitative structure–property relationship (QSPR) [13–22]. To predict drug bioactivity, ABC, Wiener, and Randic index are suitable invariants. These topological indices serve an important role in current drug design by connecting molecular structure and biological activity. Their predictive capability speeds up drug discovery, improves knowledge of structure–activity correlations, and aids in the development of safer, more effective therapies. In this article, the author calculated Tds for CRC drugs. Correspondingly, CRC treatment molecular graphs are meticulously studied with Tds and imposed QSPR (modals). Calculated results on CRC drugs depict QSPR, and Tds have a good relation. Siddiqui et al. [23] made a contribution toward titanium that mathematical modeling gives us a proficient method for changing over an issue from an application region such as science, physical science, and science into a numerical system. Titanium exists in four kinds of oxides, and various applications exist in textures, papers, food varieties, and medication. Titanium dioxide is a synthetically steady and climate-well-disposed oxide that exists in unmistakable translucent stages such as rutile, brookite, anatase, baddeleyite, columbite, and fluorite. Research on novel drugs in the treatment of CRC was discussed by Havare [13] and gave suggestion that drug discovery needs huge costs to develop and intricate processes so are efficiently predicted with this technique. QSPR modeling of blood cancer drugs was investigated by Nasir et al. [14] and observed as a suitable model for it. Being efficient and wide spread, QSPR studies for various Tds for drug structures inspired to investigate CRC drugs. The objective is to thoroughly investigate in implementing Tds to probe properties and its QSPR analysis on CRC molecular graphs in healing management. Diabetes disease drugs were discussed by Parveen et al. [15]. They impose degree base Tds with the aid of regression analysis and crafted well-developed model for RA disease. Synthetic organizations of silicate and hexagon are very much portrayed by Kulli, Chaluvaraju, and Asha [16] and made a near investigation of the designs. The deep behavior of the networks can be better understood with the help of these findings. A computational base method is applied by Adnan et al. [17] for unequivocal degree and distance-based Tds for few organizations. HIV is a deadly sickness all through the world since it has no legitimate fix to date whereas medication preliminaries are finished to battle the illness, and a proper QSPR model is carried out by Farooq et al. [18]. The QSPR illustration of heart-related drugs is observed in [19], and Bondy and Murty [20] achieved curvilinear QSPR learning of blood medicines and demonstrated right model for it. Siddiqui et al. [21] track down multiplicative Zagreb indices and found useful results of some graphs. Molecular graphs of rheumatoid arthritis worked out with topological descriptors in [24]. Drugs for vitiligo where results are provided to beautifully illuminate the subject [25]. Khan et al. [26] discussed in depth QSPR study on bladder cancer drugs. The skin cancer halts all over the world. The investigation of drugs aims to thoroughly explore and create new medications in a highly effective manner [27]. Zaman et al. [28] computed valuable formulas, and results are effective in QSPR analysis. Wang et al. [29] mentioned cancer therapy. Each year, this sickness affects up to 10 million people worldwide. Researches on molecular graphs Tds and cancer-treating drugs via QSPR correlate the physical properties. The author's deliberate study looks at some drugs that are efficiently employed in CRC therapy. The work on current study depends on Tds on many chemicals. In this scenario, the author studied degree-based topological descriptors on CRC drugs.

## 2. Materials and Methods

The molecular graphs of medications for CRC treatment drugs have been examined in this paper. Approaches such as edges partitioning, vertex partitioning, and computational methods are used to derive the topological indices of pharmaceuticals. Graph *G*(*V*, *E*) is simple and connected. The *V*(*G*) and *E*(*G*) represent vertex and edge sets, respectively. *d*_*u*_ denotes the degree of vertex. We implement the following Tds by Shigehalli and Kanabur [30]:

(1) $$\begin{array}{c} \text{SK}\left(G\right)=\underset{uv\in E\left(G\right)}{\sum }\frac{d_{u}+d_{v}}{2}, \end{array}$$

(2) $$\begin{array}{c} {\text{SK}}_{1}\left(G\right)=\underset{uv\in E\left(G\right)}{\sum }\frac{d_{u}d_{v}}{2}, \end{array}$$

(3) $$\begin{array}{c} {\text{SK}}_{2}\left(G\right)=\underset{uv\in E\left(G\right)}{\sum }{\left(\frac{d_{u}+d_{v}}{2}\right)}^{2}. \end{array}$$

KCD indices are defined by Mirajkar and Morajkar [31] as follows:

(4) $$\begin{array}{c} \text{KCD}1\left(G\right)=\underset{uv\in E\left(G\right)}{\sum }\left(d_{u}+d_{v}+d_{e}\right), \end{array}$$

(5) $$\begin{array}{c} \text{KCD}2\left(G\right)=\underset{uv\in E\left(G\right)}{\sum }\left(d_{u}d_{v}\right).d_{e}, \end{array}$$

where *d*_*e*_ = *d*_*u*_ + *d*_*v*_ − 2

Gourava indices [32] are as follows:

(6) $$\begin{array}{c} {\text{GO}}_{1}\left(G\right)=\underset{uv\in E\left(G\right)}{\sum }\left[\left(d_{u}+d_{v}\right)+d_{u}d_{v}\right], \end{array}$$

(7) $$\begin{array}{c} {\text{GO}}_{2}\left(G\right)=\underset{uv\in E\left(G\right)}{\sum }\left[\left(d_{u}+d_{v}\right)d_{u}d_{v}\right]. \end{array}$$

Randic index [33] is as follows:

(8) $$\begin{array}{c} \text{RA}\left(G\right)=\underset{uv\in E\left(G\right)}{\sum }\sqrt{\frac{1}{d_{u}d_{v}}}. \end{array}$$

GA index [34] is as follows:

(9) $$\begin{array}{c} \text{GA}\left(G\right)=\underset{uv\in E\left(G\right)}{\sum }\frac{2\sqrt{d_{u}d_{v}}}{d_{u}+d_{v}}. \end{array}$$

Harmonic index [35] of *G* is as follows:

(10) $$\begin{array}{c} H\left(G\right)=\underset{uv\in E\left(G\right)}{\sum }\frac{2}{d_{u}+d_{v}}. \end{array}$$

Hyper-Zagreb index [36] is as follows:

(11) $$\begin{array}{c} \text{HM}\left(G\right)=\underset{uv\in E\left(G\right)}{\sum }{\left(d_{u}+d_{v}\right)}^{2}. \end{array}$$

Forgotten index [37] is as follows:

(12) $$\begin{array}{c} F\left(G\right)=\underset{uv\in E\left(G\right)}{\sum }\left[{\left(d_{u}\right)}^{2}+{\left(d_{v}\right)}^{2}\right]. \end{array}$$

Zagreb indices [38] are as follows:

(13) $$\begin{array}{c} M_{1}\left(G\right)=\underset{uv\in E\left(G\right)}{\sum }d_{u}+d_{v}, \end{array}$$

(14) $$\begin{array}{c} M_{2}\left(G\right)=\underset{uv\in E\left(G\right)}{\sum }d_{u}.d_{v}. \end{array}$$

ABC index [39] *G* is as follows:

(15) $$\begin{array}{c} \text{ABC}\left(G\right)=\underset{uv\in E\left(G\right)}{\sum }\sqrt{\frac{d_{u+}d_{v}-2}{d_{u}d_{v}}}. \end{array}$$

Metastatic cancer of the colon which is treated with irinotecan is an antineoplastic enzyme inhibitor. This exhibits chemical formula C_33_H_38_N_4_O_6_. Antineoplastic is another enzyme inhibitor, and irinotecan is primarily used to treat CRC. In addition, it can be administered for adults with locally advanced rectal cancer prior to surgery. Capecitabine is recommended for treating cancer. It can also be used in combination with docetaxel when the illness has progressed after receiving anthracycline-containing chemotherapy in the past. Capecitabine is for the treatment of adult patients with unresectable or metastatic gastric, esophageal, or gastroesophageal junction. C_4_H_3_FN_2_O_2_ is its chemical composition. A pyrimidine compound exhibiting antimetabolite properties targeting tumors. By preventing thymidylate synthetase from converting deoxyuridylic acid to thymidylic acid, it prevents DNA synthesis. Leucovorin is a folate analog that is used to treat megaloblastic anemia and CRC. Despite this difference in activity, leucovorin and levoleucovorin are both used as folate analogs to prevent the harmful effects of folic acid antagonists such as methotrexate, which function by inhibiting the enzyme dihydrofolate reductase. They are suggested for use as rescue therapy after the administration of high doses of methotrexate in the treatment of osteosarcoma or for reducing the toxicity brought on by unintentional overdosage of folic acid antagonists. The use of tipiracil hydrochloride is recommended for the treatment of metastatic CRC that has already received treatment with chemotherapy which includes fluoropyrimidine, oxaliplatin, and irinotecan. Metastatic gastrointestinal stromal tumors, hepatocellular carcinoma, and metastatic CRC are all conditions that regorafenib is used to treat. C_21_H_15_ClF_4_N_4_O_3_ is the drug's chemical formula. Multiple kinases are inhibited by the oral medication regorafenib. Advanced gastrointestinal tumors, hepatocellular carcinoma, and metastatic CRC are all treated with it. In April 2017, regorafenib received approval for the treatment of hepatocellular carcinoma. Previous research on tuberculosis, breast cancer, bladder cancer, and QSPR analysis of various Tds for various drugs instigated to work CRC. The author looks into the relation of molecular graph Tds and its QSPR modeling of CRC which is suggested in therapeutic management.

## 3. Quantitative Structure Analysis and Regression Models

QSPR analysis is a computational method for predicting a chemical compound's properties or activities based on its molecular structure. The author used degree-based topological metrics and regression models to assess and forecast the efficacy of new medications for CRC illnesses. The structures of these drugs are given in Figure 1. The study of the QSPR revealed a strong connection between TIs and the physiochemical properties of drugs under investigation. In Tables 1 and 2, the author has tabulated calculations of the above Tds and physicochemical properties of molecular structures, respectively. This will show regression models of worked analysis.

### 3.1. Topological Descriptors Calculation

Let *G* is a graph of tipiracil hydrochloride with edge partitions. |*E*_1,3_| = 4, |*E*_2,2_| = 2, |*E*_3,3_| = 3, |*E*_2,3_| = 8. By applying equations (1)–(15), we get the results as

(16) $$\begin{array}{c} {\text{GO}}_{1}\left(G\right)=4\left[\left(1+3\right)+1\times 3\right]+2\left[\left(2+2\right)+2\times 2\right]+8\left[\left(2+3\right)+2\times 3\right]+3\left[\left(3+3\right)+3\times 3\right]=177,\\ {\text{GO}}_{2}\left(G\right)=4\left[\left(1+3\right)\times 1\times 3\right]+2\left[\left(2+2\right)\times 2\times 2\right]+8\left[\left(2+3\right)\times 2\times 3\right]+3\left[\left(3+3\right)\times 3\times 3\right]=482,\\ {\text{KCD}}_{1}\left(G\right)=4\left[\left(1+3\right)+2\right]+2\left[\left(2+2\right)+2\right]+8\left[\left(2+3\right)+3\right]+3\left[\left(3+3\right)+4\right]=130,\\ {\text{KCD}}_{2}\left(G\right)=4\left[\left(1+3\right)\times 2\right]+2\left[\left(2+2\right)\times 2\right]+8\left[\left(2+3\right)\times 3\right]+3\left[\left(3+3\right)\times 4\right]=240,\\ F\left(G\right)=4\left[{\left(1\right)}^{2}+{\left(3\right)}^{2}\right]+2\left[{\left(2\right)}^{2}+{\left(2\right)}^{2}\right]+8\left[{\left(2\right)}^{2}+{\left(3\right)}^{2}\right]+3\left[{\left(3\right)}^{2}+{\left(3\right)}^{2}\right]=214,\\ \text{HM}\left(G\right)=4{\left(1+3\right)}^{2}+2{\left(2+2\right)}^{2}+8{\left(2+3\right)}^{2}+3{\left(3+3\right)}^{2}=404,\\ M_{1}\left(G\right)=4\left(1+3\right)+2\left(2+2\right)+8\left(2+3\right)+3\left(3+3\right)=82,\\ M_{2}\left(G\right)=4\left(1+3\right)+2\left(2+2\right)+8\left(2+3\right)+3\left(3+3\right)=95,\\ H\left(G\right)=\frac{8}{1+3}+\frac{4}{2+2}+\frac{16}{2+3}+\frac{3}{3+3}=7.20,\\ \text{SK}\left(G\right)=4\frac{1+3}{2}+2\frac{2+2}{2}+8\frac{2+3}{2}+3\frac{3+3}{2}=41,\\ {\text{SK}}_{1}\left(G\right)=4\frac{1\times 3}{2}+2\frac{2\times 2}{2}+8\frac{2\times 3}{2}+3\frac{3\times 3}{2}=47.5,\\ {\text{SK}}_{2}\left(G\right)=4{\left(\frac{1+3}{2}\right)}^{2}+2{\left(\frac{2+2}{2}\right)}^{2}+8{\left(\frac{2+3}{2}\right)}^{2}+6{\left(\frac{3+3}{2}\right)}^{2}=101,\\ \text{GA}\left(G\right)=\frac{\left(8\sqrt{1\times 3}\right)}{\left(1+3\right)}+\frac{\left(4\sqrt{2\times 2}\right)}{\left(2+2\right)}+\frac{\left(16\sqrt{2\times 3}\right)}{\left(2+3\right)}+\frac{\left(3\sqrt{3\times 3}\right)}{\left(3+3\right)}=16.30,\\ \text{ABC}\left(G\right)=4\sqrt{\frac{\left(1+3-2\right)}{\left(1\times 3\right)}}+2\sqrt{\frac{\left(2+2-2\right)}{\left(2\times 2\right)}}+8\sqrt{\frac{\left(2+3-2\right)}{\left(2\times 3\right)}}+3\sqrt{\frac{\left(3+3-2\right)}{\left(3\times 3\right)}}=12.34,\\ \text{RA}\left(G\right)=4\sqrt{\frac{1}{1\times 3}}+2\sqrt{\frac{1}{2\times 2}}+8\sqrt{\frac{1}{2\times 3}}+3\sqrt{\frac{1}{3\times 3}}=7.58. \end{array}$$

Topological descriptors' calculation of other CRC drugs follows the same process and given in Tables 1 and 3.

### 3.2. Curvilinear Regression Models

**Figure 2:**
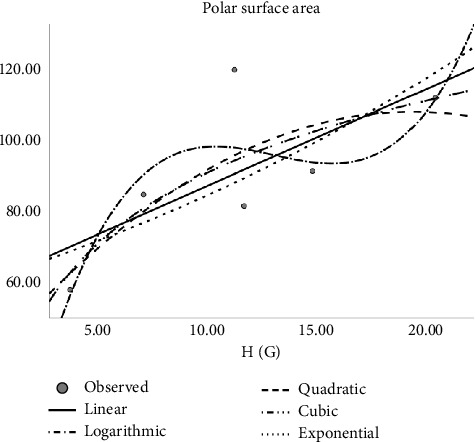
Exponential regression model of H (G) with complexity.

**Figure 3:**
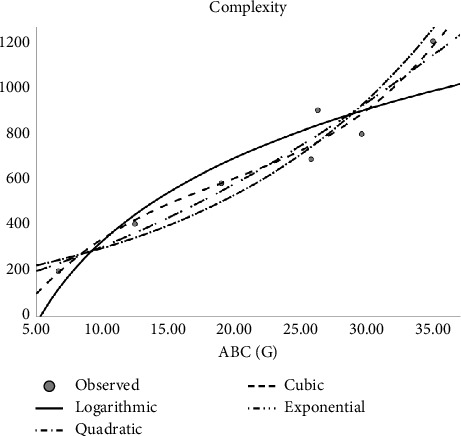
Quadratic regression model of ABC (G) with complexity.

**Figure 4:**
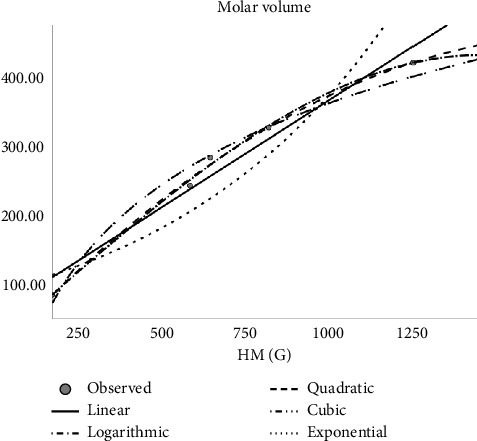
Logarithmic regression model of HM (G) with complexity.

**Figure 5:**
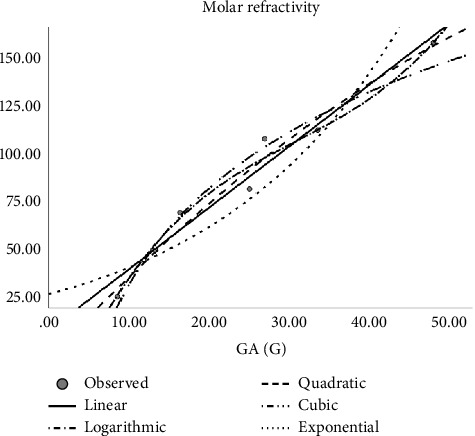
Cubic regression model of GA (G) with complexity.

Regression models are used to fit the curves. Accordingly, linear, quadratic, cubic, logarithmic, and exponential regression models are studied. The author constructed regression models of the abovementioned Tds with the physicochemical properties of molecular structures as shown in Tables 4, 5, 6, 7, 8, and 9. In the regression model table, we considered the square of the coefficient of the correlation (*R*^2^) and significance (sig). The maximum *R*^2^ is goodness of fit of the regression model, and the sig value is less than 0.05. Here, several top topological descriptors are applied as predictors to evaluate and carry out this analysis. In this study, the author tested the following equations:

- *Z* = *ae*^*t*_1_^ (exponential equation)
- *Z* = *a* ln(*t*_1_) (logarithmic equation)
- *Z* = *a* + *b*_1_*t*_1_ + *b*_2_*t*_1_^2^ (quadratic equation)
- *Z* = *a* + *b*_1_*t*_1_ + *b*_2_*t*_1_^2^ + *b*_3_*t*_1_^3^ (cubic equation)

where *Z* is the dependent variable, ‘*a*' is the regression model constant, *t*_*i*_ are independent variables, and *b*_*i*_(*i* = 1, 2, 3,…) are the coefficients for the individual descriptor. The physical properties are found at ChemSpider. Figures 2, 3, 4, and 5 depict the graph between Tds and physical properties. The author finds relation of Tds with the properties of aforementioned drugs irinotecan, capecitabine, fluorouracil, leucovorin, tipiracil hydrochloride, regorafenib, tucatinib, and bevacizumab, and it will be best calculated with the aid of QSPR modeling. The observations evaluate that *p* should be less than 0.05 and *r* is greater than 0.7. The information obtained provided the features listed in Tables 2, 4, 5, 6, 7, 8, 9, and 10 is vital, and the predicted is mentioned in Tables 11 and 12.

## 4. Results and Discussion

The study focuses on conducting QSPR analysis of the CRC drug molecules, and predictive equations were developed for the physicochemical properties using quadratic, cubic, exponential, and logarithmic regression models whereas the correlation values provided in Tables 3, 4, 5, 6, 7, 8, 9, and 10 for their models based on degree base indices are discussed below to obtain the clear picture. In this section, we discuss the effectiveness of our models in predicting the properties of the cancer drugs by imposing model equation in Section 3.2. In this study, the proposed quadratic regression models based on SK_1_(G), GO_1_(G), and *F*(G) are evaluated under the following:

(17) $$\begin{array}{c} P=-0.0012\left[{\text{SK}}_{1}^{2}\right]+0.5935\left[{\text{SK}}_{1}\right]+0.2019,\\ P=-0.0001\left[{\text{GO}}_{1}^{2}\right]+0.1587\left[{\text{GO}}_{1}\right]-0.1296,\\ P=-0.0001\left[F^{2}\right]+0.1286\left[F\right]+0.3231. \end{array}$$

It is evident that the regression models exhibit higher correlation values than those listed above, as shown below:

(18) $$\begin{array}{c} P=1.1631\left[{\text{SK}}_{1}\right]-11.4970+0.0\left[{\text{SK}}_{1}^{3}\right]-0.0086\left[{\text{SK}}_{1}^{2}\right],\\ P=0.3382\left[{\text{GO}}_{1}\right]-13.7868+0.0\left[{\text{GO}}_{1}^{3}\right]+-0.0007\left[{\text{GO}}_{1}^{2}\right],\\ P=+0.2972\left[F\right]-15.2861+0.0\left[F^{3}\right]-0.0005\left[F^{2}\right]. \end{array}$$

The logarithmic regression models produced in our study show moderate correlation values than those listed above. In addition, RA is a good predictor.

(19) $$\begin{array}{c} P=-74.7683+26.6097\;\log\left[{\text{SK}}_{1}\right],\\ P=-113.5295+27.2791\;\log\left[{\text{GO}}_{1}\right],\\ P=-119.0453+27.3372\;\log\left[F\right]. \end{array}$$

The most suitable exponential regression models here are identified as follows:

(20) $$\begin{array}{c} \log\left(P\right)=2.5494+0.0120\left[{\text{SK}}_{1}\right],\\ \log\left(P\right)=2.5121+0.0034\left[{\text{GO}}_{1}\right],\\ \log\left(P\right)=2.5111+0.0028\left[F\right]. \end{array}$$

The proposed quadratic regression models based on KCD_2_(G), RA(G), and ABC(G) are evaluated under

(21) $$\begin{array}{c} \text{MV}=-0.0003\left[{\text{KCD}}_{1}^{2}\right]+0.6430\left[{\text{KCD}}_{2}\right]+76.4820,\\ \text{MV}=-0.3737\left[{\text{RA}}^{2}\right]+23.9987\left[\text{RA}\right]+43.8578,\\ \text{MV}=-0.1433\left[{\text{ABC}}^{2}\right]+14.5494\left[\text{ABC}\right]+50.2830. \end{array}$$

The cubic models derived from our considered topological indices that provide more accurate predictions than those mentioned above are presented as follows:

(22) $$\begin{array}{c} \text{MV}=2.9123\left[{\text{KCD}}_{2}\right]-161.0176+0.0\left[{\text{KCD}}_{1}^{3}\right]-0.0061\left[{\text{KCD}}_{1}^{2}\right],\\ \text{MV}=143.1672\left[\text{RA}\right]-331.7737+0.2812\left[{\text{RA}}^{3}\right]-10.9840\left[{\text{RA}}^{2}\right],\\ \text{MV}=83.0292\left[\text{ABC}\right]-302.4526+0.0598\left[{\text{ABC}}^{3}\right]-3.8598\left[{\text{ABC}}^{2}\right]. \end{array}$$

The logarithmic regression models produced in our study show moderate correlation values than those listed above. In addition, RA is a good predictor.

(23) $$\begin{array}{c} \text{MV}=-544.1135+139.9396\;\log\left[{\text{KCD}}_{2}\right],\\ \text{MV}=-112.2471+160.4289\;\log\left[\text{RA}\right],\\ \text{MV}=-175.2019+155.7585\;\log\left[\text{ABC}\right]. \end{array}$$

The most suitable exponential regression models identified here are as follows:

(24) $$\begin{array}{c} \log\left(\text{MV}\right)=4.8510+0.0017\left[{\text{KCD}}_{2}\right],\\ \log\left(\text{MV}\right)=4.6745+0.0686\left[\text{RA}\right],\\ \log\left(\text{MV}\right)=4.7202+0.0401\left[\text{ABC}\right]. \end{array}$$

## 5. Conclusions

The author observed correlation coefficients between the Td and some physical features of drugs applied to treat CRC that depicts how fine the said descriptors serve as interpreters. Descriptors (Tds) and QSPR analysis especially in the context of pharmaceutical and medical applications forecast the physical properties. Notably, molar refractivity, molar volume, polarity, and complexity are reliable and indicators for these predictions. However, the estimation of boiling point and polar surface area is less dependable. The medications used to treat CRC are the subject of this study. Pharmaceutical industry researchers and chemists may find use in the presented measurements provided in this work. Designing novel medications may benefit from these recent findings. Based on the discovered correlations, scientists can use the correlation coefficients of various medications to find the right composition for creating new drugs for emerging disorders. Multiple topological descriptors can be incorporated in unique ways to address inequalities by using a unified methodology. This article's future recommendation suggests employing diverse forms to calculate the index's extreme values.

## Figures and Tables

**Table 1:** Topological descriptors (SK, SK_1_, SK_2_, GO_1_, GO_2_, KCD_1_, KCD_2_) of CRC drugs.

Drugs	SK	SK_1_	SK_2_	GO_1_	GO_2_	KCD_1_	KCD_2_
Irinotecan	123	153	318.5	552	1636	394	782
Capecitabine	62	72	152.5	268	736	196	362
Fluorouracil	21	23	50	88	226	66	116
Leucovorin	86	99.5	210.5	371	1004	272	498
Tipiracil hydrochloride	41	47.5	101	177	482	130	240
Regorafenib	85	97.5	211	365	994	270	504
Tucatinib	101	119.5	252.5	441	1212	322	606
Bevacizumab	46	51.5	109.5	195	510	144	254

**Table 2:** Logarithmic and quadratic regression models for molar volume (cm^3^).

Molar volume				
Regression model	Molecular descriptor	*R* ^2^	F	Sig
Logarithmic	SK (G)	0.972	103.177	0.002
	SK_1_ (G)	0.976	122.284	0.002
	SK_2_ (G)	0.976	122.094	0.002
	GO_1_ (G)	0.974	114.33	0.002
	GO_2_ (G)	0.969	93.57	0.002
	M_1_ (G)	0.972	103.177	0.002
	M_2_ (G)	0.976	122.284	0.002
	KCD_1_ (G)	0.973	107.11	0.002
	KCD_2_ (G)	0.978	135.197	0.001
	ABC (G)	0.965	83.395	0.003
	RA (G)	0.961	74.104	0.003
	F (G)	0.975	119.185	0.002
	HM (G)	0.976	122.094	0.002

Quadratic	SK (G)	0.992	128.406	0.008
	SK_1_ (G)	0.991	115.606	0.009
	SK_2_ (G)	0.993	146.931	0.007
	GO_1_ (G)	0.992	124.061	0.008
	GO_2_ (G)	0.991	116.181	0.009
	M_1_ (G)	0.992	128.406	0.008
	M_2_ (G)	0.991	115.606	0.009
	KCD_1_ (G)	0.992	126.676	0.008
	KCD_2_ (G)	0.994	155.744	0.006
	ABC (G)	0.994	156.128	0.006
	RA (G)	0.997	290.7	0.003
	F (G)	0.994	174.768	0.006
	HM (G)	0.993	146.931	0.007

**Table 3:** Topological descriptors (ABC, RA, GA, M_1_, M_2_, H, HM, and F) of CRC drugs.

Drugs	ABC	RA	GA	M_1_	M_2_	H	HM	F
Irinotecan	34.57	20.82	47.78	246	306	20.20	1274	662
Capecitabine	18.80	11.85	24.96	124	144	11.27	610	322
Fluorouracil	6.65	4.20	8.52	42	46	3.93	200	108
Leucovorin	25.98	16.19	34.66	172	199	15.47	842	444
Tipiracil hydrochloride	12.34	7.58	16.30	82	95	7.20	404	214
Regorafenib	25.48	15.56	33.41	170	195	14.75	844	454
Tucatinib	29.26	17.42	39.87	202	239	16.90	1010	532
Bevacizumab	14.46	9.49	19.19	92	103	9.03	438	232

**Table 4:** Cubic and exponential regression models for polarity (cm^3^).

Polarity				
Regression model	Molecular descriptor	*R* ^2^	F	Sig
Cubic	SK (G)	0.999	1149.679	0.000
	SK_1_ (G)	0.999	1308.204	0.000
	SK_2_ (G)	0.999	845.706	0.000
	GO_1_ (G)	0.999	1260.645	0.000
	GO_2_ (G)	0.999	871.324	0.000
	M_1_ (G)	0.999	1149.679	0.000
	M_2_ (G)	0.999	1308.204	0.000
	KCD_1_ (G)	0.999	1159.723	0.000
	KCD_2_ (G)	0.999	684.923	0.000
	ABC (G)	0.999	683.200	0.000
	RA (G)	0.995	202.808	0.000
	F (G)	0.998	493.268	0.000
	HM (G)	0.999	845.706	0.000

Exponential	SK (G)	0.914	53.243	0.001
	SK_1_ (G)	0.888	39.521	0.001
	SK_2_ (G)	0.896	43.225	0.001
	GO_1_ (G)	0.900	45.177	0.001
	GO_2_ (G)	0.919	58.866	0.001
	M_1_ (G)	0.914	53.243	0.001
	M_2_ (G)	0.888	39.521	0.001
	KCD_1_ (G)	0.910	50.766	0.001
	KCD_2_ (G)	0.883	37.917	0.002
	ABC (G)	0.935	71.701	0.000
	RA (G)	0.941	50.321	0.000
	F (G)	0.903	46.633	0.001
	HM (G)	0.896	43.225	0.001

**Table 5:** Cubic and exponential regression models for molar volume (cm^3^).

Molar volume				
Regression model	Molecular descriptor	*R* ^2^	F	Sig
Cubic	SK (G)	0.994	54.83	0.099
	SK_1_ (G)	0.993	44.569	0.11
	SK_2_ (G)	0.995	61.453	0.093
	GO_1_ (G)	0.993	49.408	0.104
	GO_2_ (G)	0.992	43.669	0.111
	M_1_ (G)	0.994	54.83	0.099
	M_2_ (G)	0.993	44.569	0.11
	KCD_1_ (G)	0.994	55.175	0.099
	KCD_2_ (G)	0.995	64.835	0.091
	ABC (G)	0.995	68.533	0.089
	RA (G)	0.997	101.592	0.073
	F (G)	0.996	85.03	0.08
	HM (G)	0.995	61.453	0.093

Exponential	SK (G)	0.911	30.621	0.012
	SK_1_ (G)	0.881	22.218	0.018
	SK_2_ (G)	0.893	25.15	0.015
	GO_1_ (G)	0.895	25.577	0.015
	GO_2_ (G)	0.915	32.384	0.011
	M_1_ (G)	0.911	30.621	0.012
	M_2_ (G)	0.881	22.218	0.018
	KCD_1_ (G)	0.907	29.154	0.012
	KCD_2_ (G)	0.881	22.313	0.018
	ABC (G)	0.936	44.215	0.007
	RA (G)	0.949	56.038	0.005
	F (G)	0.904	28.143	0.013
	HM (G)	0.893	25.15	0.015

**Table 6:** Logarithmic and quadratic regression models for molar refractivity (cm^3^).

Molar refractivity				
Regression model	Molecular descriptor	*R* ^2^	F	Sig
Logarithmic	SK (G)	0.959	115.999	0.000
	SK_1_ (G)	0.963	131.414	0.000
	SK_2_ (G)	0.962	125.202	0.000
	GO_1_ (G)	0.962	125.218	0.000
	GO_2_ (G)	0.957	110.093	0.000
	M_1_ (G)	0.959	115.999	0.000
	M_2_ (G)	0.963	131.414	0.000
	KCD_1_ (G)	0.960	119.025	0.000
	KCD_2_ (G)	0.963	129.440	0.000
	ABC (G)	0.951	96.0680	0.000
	RA (G)	0.943	82.9300	0.000
	F (G)	0.959	118.437	0.000
	HM (G)	0.962	125.202	0.000

Quadratic	SK (G)	0.980	100.494	0.000
	SK_1_ (G)	0.982	107.74	0.000
	SK_2_ (G)	0.980	98.069	0.000
	GO_1_ (G)	0.981	105.211	0.000
	GO_2_ (G)	0.981	101.711	0.000
	M_1_ (G)	0.980	100.494	0.000
	M_2_ (G)	0.982	107.74	0.000
	KCD_1_ (G)	0.981	100.627	0.000
	KCD_2_ (G)	0.979	95.52	0.000
	ABC (G)	0.979	91.774	0.000
	RA (G)	0.976	81.483	0.001
	F (G)	0.978	88.229	0.000
	HM (G)	0.980	98.069	0.000

**Table 7:** Cubic and exponential regression models for molar refractivity (cm^3^).

Molar refractivity				
Regression model	Molecular descriptor	*R* ^2^	F	Sig
Cubic	SK (G)	0.982	53.956	0.004
	SK_1_ (G)	0.982	54.327	0.004
	SK_2_ (G)	0.981	50.373	0.005
	GO_1_ (G)	0.982	54.01	0.004
	GO_2_ (G)	0.982	54.016	0.004
	M_1_ (G)	0.982	53.956	0.004
	M_2_ (G)	0.982	54.327	0.004
	KCD_1_ (G)	0.982	53.62	0.004
	KCD_2_ (G)	0.980	48.449	0.005
	ABC (G)	0.982	55.083	0.004
	RA (G)	0.980	48.388	0.005
	F (G)	0.979	46.589	0.005
	HM (G)	0.981	50.373	0.005

Exponential	SK (G)	0.862	31.229	0.003
	SK_1_ (G)	0.837	25.606	0.004
	SK_2_ (G)	0.844	27.067	0.003
	GO_1_ (G)	0.849	28.058	0.003
	GO_2_ (G)	0.867	32.703	0.002
	M_1_ (G)	0.862	31.229	0.003
	M_2_ (G)	0.837	25.606	0.004
	KCD_1_ (G)	0.858	30.252	0.003
	KCD_2_ (G)	0.831	24.641	0.004
	ABC (G)	0.883	37.599	0.002
	RA (G)	0.889	40.231	0.001
	F (G)	0.850	28.309	0.003
	HM (G)	0.844	27.067	0.003

**Table 8:** Logarithmic and quadratic regression models for complexity (cm^3^).

Complexity				
Regression model	Molecular descriptor	*R* ^2^	F	Sig
Logarithmic	SK (G)	0.863	31.572	0.002
	SK_1_ (G)	0.875	34.857	0.002
	SK_2_ (G)	0.869	33.203	0.002
	GO_1_ (G)	0.870	33.438	0.002
	GO_2_ (G)	0.864	31.863	0.002
	M_1_ (G)	0.863	31.572	0.002
	M_2_ (G)	0.875	34.857	0.002
	KCD_1_ (G)	0.864	31.780	0.002
	KCD_2_ (G)	0.872	34.068	0.002
	ABC (G)	0.854	29.235	0.003
	RA (G)	0.854	29.235	0.003
	F (G)	0.864	31.668	0.002
	HM (G)	0.869	33.203	0.002

Quadratic	SK (G)	0.935	28.791	0.004
	SK_1_ (G)	0.941	32.106	0.003
	SK_2_ (G)	0.936	29.074	0.004
	GO_1_ (G)	0.939	30.84	0.004
	GO_2_ (G)	0.942	32.207	0.003
	M_1_ (G)	0.935	28.791	0.004
	M_2_ (G)	0.941	32.106	0.003
	KCD_1_ (G)	0.934	28.24	0.004
	KCD_2_ (G)	0.935	28.812	0.004
	ABC (G)	0.936	29.27	0.004
	RA (G)	0.951	38.662	0.002
	F (G)	0.929	26.32	0.005
	HM (G)	0.936	29.074	0.004

**Table 9:** Cubic and exponential regression models for complexity (cm^3^).

Complexity				
Regression model	Molecular descriptor	*R* ^2^	F	Sig
Cubic	SK (G)	0.947	18.036	0.020
	SK_1_ (G)	0.950	18.836	0.019
	SK_2_ (G)	0.946	17.611	0.021
	GO_1_ (G)	0.949	18.571	0.019
	GO_2_ (G)	0.952	19.938	0.017
	M_1_ (G)	0.947	18.036	0.020
	M_2_ (G)	0.950	18.836	0.019
	KCD_1_ (G)	0.946	17.602	0.021
	KCD_2_ (G)	0.945	17.221	0.021
	ABC (G)	0.952	19.884	0.018
	RA (G)	0.966	28.247	0.011
	F (G)	0.943	16.470	0.023
	HM (G)	0.946	17.611	0.021
Exponential	SK (G)	0.912	51.737	0.001
	SK_1_ (G)	0.896	43.194	0.001
	SK_2_ (G)	0.899	44.391	0.001
	GO_1_ (G)	0.904	47.151	0.001
	GO_2_ (G)	0.92	57.557	0.001
	M_1_ (G)	0.912	51.737	0.001
	M_2_ (G)	0.896	43.194	0.001
	KCD_1_ (G)	0.908	49.487	0.001
	KCD_2_ (G)	0.889	39.987	0.001
	ABC (G)	0.928	64.675	0.000
	RA (G)	0.942	81.073	0.000
	F (G)	0.900	44.963	0.001
	HM (G)	0.899	44.391	0.001

**Table 10:** Logarithmic and quadratic regression models for polarity (cm^3^).

Polarity				
Regression model	Molecular descriptor	*R* ^2^	F	Sig
Logarithmic	SK (G)	0.956	107.600	0.000
	SK_1_ (G)	0.959	118.303	0.000
	SK_2_ (G)	0.959	116.966	0.000
	GO_1_ (G)	0.958	114.361	0.000
	GO_2_ (G)	0.953	101.427	0.000
	M_1_ (G)	0.956	107.600	0.000
	M_2_ (G)	0.959	118.303	0.000
	KCD_1_ (G)	0.957	110.387	0.000
	KCD_2_ (G)	0.960	121.463	0.000
	ABC (G)	0.947	89.8870	0.000
	RA (G)	0.940	77.9960	0.000
	F (G)	0.958	114.203	0.000
	HM (G)	0.959	116.966	0.000

Quadratic	SK (G)	0.999	2236.871	0.000
	SK_1_ (G)	0.998	1152.827	0.000
	SK_2_ (G)	0.998	1224.954	0.000
	GO_1_ (G)	0.999	1637.881	0.000
	GO_2_ (G)	0.999	1659.673	0.000
	M_1_ (G)	0.999	2236.871	0.000
	M_2_ (G)	0.998	1152.827	0.000
	KCD_1_ (G)	0.999	2184.127	0.000
	KCD_2_ (G)	0.998	869.0050	0.000
	ABC (G)	0.998	1087.637	0.000
	RA (G)	0.995	376.6590	0.000
	F (G)	0.998	937.4390	0.000
	HM (G)	0.998	1224.954	0.000

**Table 11:** Comparison of actual versus predicted values for molar volume.

Drugs	Polarity	Quadratic	Cubic	Logarithmic	Exponential
*Predicted values withSK*_1_ *(G) index*					
Irinotecan	63.1	62.221	63.666	80.089	59.09
Capecitabine	32.6	36.558	37.829	30.337	39.033
Fluorouracil	10.2	13.201	11.023	16.863	8.666
Leucovorin	46.33	47.08	46.058	42.18	47.641
Tipiracil hydrochloride		25.618	27.237	22.618	27.965
Regorafenib	44.8	46.378	45.519	41.181	47.10
Tucatinib	53.6	53.564	51.51	53.605	52.514
Bevacizumab		27.505	29.285	23.728	30.116

*Predicted values withGO*_1_ *(G) index*					
Irinotecan	63.1	62.013	63.716	58.698	78.709
Capecitabine	32.6	36.405	37.725	38.988	30.328
Fluorouracil	10.2	13.191	10.841	8.608	16.570
Leucovorin	46.33	47.251	46.013	47.859	42.860
Tipiracil hydrochloride		25.346	27.137	27.671	22.342
Regorafenib	44.8	46.668	45.566	47.414	42.005
Tucatinib	53.6	53.610	51.503	52.574	54.218
Bevacizumab		27.643	29.626	30.313	23.734

*Predicted values with F (G) index*					
Irinotecan	63.1	61.847	63.772	58.517	77.724
Capecitabine	32.6	36.150	37.662	38.815	30.178
Fluorouracil	10.2	13.585	10.949	8.951	16.637
Leucovorin	46.33	46.804	45.556	47.597	42.375
Tipiracil hydrochloride		25.379	27.342	27.645	22.345
Regorafenib	44.8	47.606	46.160	48.206	43.571
Tucatinib	53.6	53.493	51.259	52.541	54.132
Bevacizumab		27.262	29.425	29.853	23.492

**Table 12:** Comparison of actual versus predicted values for polarity.

Drugs	Molar volume	Quadratic	Cubic	Logarithmic	Exponential
*Predicted values withKCD*_2_ *(G) index*					
Irinotecan	416.800	395.153	423.869	388.144	477.587
Capecitabine	240.500	269.777	297.330	280.361	235.334
Fluorouracil	84.600	147.013	101.597	121.102	155.472
Leucovorin		322.004	304.456	324.995	295.946
Tipiracil hydrochloride		213.449	246.233	222.845	191.602
Regorafenib	323.700	324.052	304.513	326.671	298.954
Tucatinib	339.000	355.544	313.556	352.463	355.018
Bevacizumab		220.368	255.807	230.779	196.176

*Predicted values with RA (G) index*					
Irinotecan	416.800	381.524	425.656	374.801	447.450
Capecitabine	240.500	275.767	290.305	284.386	241.740
Fluorouracil	84.600	138.060	96.606	117.982	142.988
Leucovorin		334.445	300.409	334.450	325.630
Tipiracil hydrochloride		204.297	244.809	212.704	180.326
Regorafenib	323.700	326.801	295.958	328.083	311.848
Tucatinib	339.000	348.515	315.605	346.198	354.316
Bevacizumab		237.950	278.012	248.756	205.587

*Predicted values with ABC (G) index*					
Irinotecan	416.800	381.988	425.618	376.648	448.140
Capecitabine	240.500	273.160	291.625	281.771	238.252
Fluorouracil	84.600	140.699	96.585	119.901	146.434
Leucovorin		331.548	298.029	332.154	317.657
Tipiracil hydrochloride		208.000	246.736	216.195	183.925
Regorafenib	323.700	327.960	296.441	329.128	311.358
Tucatinib	339.000	353.304	320.434	350.673	362.265
Bevacizumab		230.702	271.892	240.889	200.229

## Data Availability

All the data are available inside the manuscript, and there are no hidden data.
